# Cognitive Correlates of Emotional Dispositions: Differentiating Trait Sadness and Trait Anger via Attributional Style and Helplessness

**DOI:** 10.3390/bs15101401

**Published:** 2025-10-15

**Authors:** Seunghee Han

**Affiliations:** Department of Business Administration, Chung-Ang University, Seoul 06974, Republic of Korea; shan@cau.ac.kr

**Keywords:** trait sadness, discrete emotions, trait anger, cognitive appraisal, attribution, helplessness, stability, globality, personality

## Abstract

While sadness and anger are distinct emotional states, the cognitive traits that differentiate people prone to one versus the other are not well understood. This research tested whether the cognitive signatures of state emotions extend to the trait level. Across two studies, we developed and validated a new Trait Sadness Scale (TSS) and used it to compare the cognitive responses of a sadness-prone group (high sadness, low anger) and an anger-prone group (high anger, low sadness) to ambiguous negative events. Contrary to predictions from state emotion theories, the groups did not differ in their causal attribution patterns (i.e., who they blamed). However, key cognitive differences did emerge: the sadness-prone group reported significantly greater helplessness, an effect specific to interpersonal contexts, and appraised the causes of negative events as more stable and global. These findings reveal a dissociation between state- and trait-level cognition, suggesting that emotional dispositions are differentiated not by simple patterns of blame, but by a more complex interplay of context-dependent appraisals of control and a pessimistic explanatory style.

## 1. Introduction

Individual differences in emotional experience are a cornerstone of personality science. While it is widely accepted that people have stable tendencies to experience certain emotions more frequently and intensely than others, the cognitive architecture of these emotional dispositions remains a critical area of inquiry. Among the negative emotions, anger and sadness represent two of the most fundamental and socially significant responses to adverse events. A substantial body of evidence indicates they are distinct constructs with unique signatures, challenging earlier models that grouped them under a single “Negative Affectivity” construct (Watson & Clark, 1992). Yet, an asymmetry exists in their measurement: while robust instruments for trait anger are well-established, such as the State–Trait Anger Expression Inventory (Spielberger et al., 1983), a comparable, dedicated measure for trait sadness—a dispositional tendency to experience sadness—has been notably absent.

This absence of a nuanced measure for trait sadness is a significant methodological gap. While broad measures of affect, such as the Positive and Negative Affect Schedule Expanded Form (PANAS-X; Watson & Clark, 1994), include subscales for sadness, these are typically limited to a few general adjectives (e.g., sad, blue, and downhearted). Such indices are useful for capturing general affective tone but lack the theoretical depth to assess the multi-faceted nature of a personality trait. They do not adequately capture the cognitive appraisals (e.g., helplessness), behavioral tendencies (e.g., withdrawal), and somatic experiences that constitute a stable disposition toward sadness. This effort to develop a more nuanced, dedicated trait measure for sadness parallels recent advancements in the study of anger, where researchers have also moved toward developing comprehensive scales that capture the specific components of the trait (e.g., Umbra & Fasbender, 2025).

A primary motivation for developing this tool is to investigate a central question in emotion science: whether the unique cognitive signatures of anger and sadness, well-documented at the transient state level, also manifest as stable patterns at the dispositional trait level. Cognitive appraisal theory provides a powerful framework for this question. The theory posits that anger and sadness are differentiated by appraisals of accountability and personal control. At the state level, anger is typically elicited when a negative event is attributed to another’s violation (other accountability), whereas sadness arises when the cause is perceived as circumstantial (situational accountability) (e.g., Bodenhausen et al., 1994; Keltner et al., 1993). Likewise, anger is associated with an appraisal of high personal control or agency, while sadness is characterized by appraisals of helplessness and a perceived lack of control. (e.g., Bernbau et al., 1995; Tiedens, 2001).

The current study tests whether these appraisal patterns extend from momentary states to enduring traits. If trait emotions function as chronic cognitive filters (Damasio, 1994) just like state emotions function as situational cognitive filters, a twofold hypothesis can be formed. First, a dispositional tendency toward anger will be associated with a cognitive bias for other accountability, whereas a tendency toward sadness will be associated with a bias for situational accountability. Second, a disposition toward sadness will be uniquely linked to greater feelings of helplessness, reflecting a diminished sense of agency.

The development of a valid Trait Sadness Scale (TSS) to investigate these cognitive correlates is not merely a theoretical exercise; it also holds significant practical implications. In clinical and counseling contexts, for instance, the TSS could help individuals recognize their core emotional patterns and cognitive styles that sustain them, facilitating interventions aimed at improving coping and emotion regulation. As a research instrument, the TSS enables a more precise exploration of the downstream consequences of trait sadness on well-being, interpersonal relationships, and decision-making.

To achieve these goals, the present research unfolds in three stages. Study 1 describes the development and initial validation of the 15-item Trait Sadness Scale (TSS), establishing its three-factor structure. Study 1-2 confirms this structure in a larger, independent sample and establishes the scale’s construct validity. Finally, Study 2 provides experimental evidence for the TSS’s predictive utility by testing the core hypothesis that the cognitive signatures of differentiating state emotions extend to the trait level, shaping how individuals appraise ambiguous negative events.

## 2. Study 1-1: Development and Initial Validation of the Trait Sadness Scale (TSS)

Study 1 aimed to address a methodological gap in emotion research by developing and validating a scale to measure trait sadness. The development of this instrument was a necessary first step to empirically investigate the cognitive correlates of sadness-prone and anger-prone dispositions.

### 2.1. Method

#### 2.1.1. Item Generation and Content Validation

The scale development proceeded in two stages: item pool generation and content validity assessment.

First, an initial pool of 60 items was generated by triangulating theoretical frameworks with qualitative descriptions of lived experience. A literature review drawing on prototypical emotion scripts (e.g., Shaver et al., 1987) and cognitive appraisal theories (e.g., Roseman et al., 1994) was conducted to establish three core theoretical dimensions of trait sadness:

- Cognitive Appraisals: Tendencies to appraise situations in terms of loss, helplessness, and negative outcomes (e.g., “I often feel like things are hopeless”).
- Affective Experience: The propensity to experience the core feelings of sadness, gloom, and disappointment (e.g., “I feel downhearted quite often”).
- Motivational/Behavioral Tendencies: The inclination toward withdrawal, inaction, and expressions of distress (e.g., “When I’m upset, I prefer to be alone”).

To supplement these theoretical domains, open-ended descriptions of sadness episodes were collected from 21 psychology graduate students. This combined process yielded an initial pool of 60 items designed to tap the three dimensions above.

Next, the 60-item pool was subjected to a quantitative content validation by a panel of eleven expert judges (Ph.D. candidates and faculty in personality and counseling psychology). The judges were provided with definitions and example items for the three theoretical dimensions. For each of the 60 items, they were asked to perform two tasks:

- Relevance Rating: Rate the relevance of each item to the overarching construct of trait sadness on a 4-point scale (1 = not relevant, 2 = somewhat relevant, 3 = quite relevant, and 4 = highly relevant).
- Clarity Rating: Rate the clarity of each item’s wording on a 3-point scale (1 = unclear, 2 = needs minor revision, and 3 = very clear).

An item was retained only if it received a relevance rating of 3 or higher from at least nine of the 11 judges and had a mean clarity rating of 2.5 or higher. This rigorous, quantitative review process reduced the pool to a set of 36 preliminary items that demonstrated high content validity and clarity.

#### 2.1.2. Participants

Participants were 161 undergraduates (109 men, 52 women) at a major university. After providing written informed consent, they participated for course credit. The mean age of the sample was 22.7 years (*SD* = 4.0).

#### 2.1.3. Measures

Preliminary Trait Sadness Scale (TSS). The preliminary TSS comprised 36 items. Participants rated their agreement with each item on a 5-point scale from 1 (*strongly disagree*) to 5 (*strongly agree*). The instructions were matched to those of the Trait Anger Scale to ensure procedural consistency.

Trait Anger Scale (TAS). Trait anger was assessed using the Korean adaptation (Chon et al., 1998) of Spielberger et al.’s (1983) scale. It measures general tendency to experience angry feelings. To ensure consistency, it was administered on a 5-point scale. The scale demonstrated excellent internal consistency (Cronbach’s α = 0.87).

Differential Emotions Scale–IV (DES-IV). The tendency to experience seven basic emotions (Interest, Joy, Sadness, Anger, Contempt, Surprise, and Fear) was measured using the DES-IV (Izard et al., 1993). Items were rated on a 5-point scale. The Sadness and Anger subscales showed high internal consistency (both α = 0.85).

### 2.2. Results

#### 2.2.1. Exploratory Factor Analysis and Item Reduction

An Exploratory Factor Analysis (EFA) was conducted to determine the underlying structure of the 36 preliminary items and to finalize the scale.

Analytical Strategy. Given the ordinal nature of the 5-point Likert scale data, the EFA was performed on a polychoric correlation matrix. Parallel analysis, which compares observed eigenvalues to those from random data, clearly supported a three-factor solution consistent with our theoretical framework. We proceeded with a three-factor EFA using maximum likelihood extraction and an oblique (promax) rotation, as the underlying factors of an emotional disposition were expected to be correlated. Items were retained if they had a factor loading > 0.50, no significant cross-loadings (>0.30), and communalities > 0.30.

Factor Structure and Final Scale. This iterative process removed 21 items, resulting in a final 15-item scale with a clean three-factor structure (see Table 1). The three factors were theoretically coherent and interpreted as follows:

- Factor 1: Affective Frequency (5 items): The general propensity to feel sad, dejected, and depressed.
- Factor 2: Cognitive Reactions (5 items): Cognitive patterns associated with sadness, such as feelings of helplessness and difficulty recovering from setbacks.
- Factor 3: Somatic & Behavioral Expressions (5 items): Somatic and action tendencies, such as crying and withdrawal.

#### 2.2.2. Reliability

The final 15-item TSS demonstrated excellent internal consistency for the total scale (Cronbach’s α = 0.90). The subscales also showed good reliability: Affective (α = 0.88), Cognitive Responses (α = 0.85), and Somatic/Behavioral Responses (α = 0.86).

#### 2.2.3. Preliminary Convergent and Discriminant Validity

To provide initial evidence of validity, the TSS scores were correlated with the seven DES-IV emotion subscales and the TAS. To control for Type I error across these multiple comparisons, a Bonferroni correction was applied, setting the significance threshold at *p* < 0.007.

As shown in Table 2, the analyses provided strong support for the scale’s validity. The TSS total score showed a large, positive correlation with the DES-Sadness subscale (*r* = 0.74, *p* < 0.001), confirming convergent validity. In contrast, correlations with other negative emotions like Anger (*r* = 0.55, *p* < 0.001) and Fear (*r* = 0.46, *p* < 0.001) were significantly positive but weaker, providing preliminary evidence of discriminant validity.

The TSS also showed a moderate positive correlation with the TAS (*r* = 0.31, *p* < 0.001), indicating that the scales are related constructs under the broader umbrella of negative affectivity but remain distinct.

## 3. Study 1-2: Confirmatory Factor Analysis and Construct Validation of the TSS

This study had two primary objectives: first, to confirm the three-factor structure of the Trait Sadness Scale (TSS) in a large, diverse general population sample, and second, to establish its broader construct validity. By moving beyond the undergraduate sample in Study 1, this study provides a more robust test of the scale’s psychometric properties and the generalizability of its findings.

Specifically, the study sought to delineate the distinct personality profiles of individuals prone to sadness versus anger by examining relationships with self-esteem, self-efficacy, optimism, and depressive tendencies. It was hypothesized that trait sadness would be uniquely associated with a profile of diminished self-appraisals and greater depressive symptoms. We tested this hypothesis by conducting a Confirmatory Factor Analysis (CFA) followed by a series of correlational analyses.

### 3.1. Method

#### 3.1.1. Participants

A total of 624 adults were recruited from a general population sample in South Korea. All participants provided written informed consent in accordance with Institutional Review Board (IRB) guidelines. The sample consisted of 319 men, 295 women, and 10 participants who did not report their gender. The mean age was 28 years (*SD* = 4.7).

#### 3.1.2. Measures

Trait Sadness Scale (TSS). The final 15-item, three-factor scale developed in Study 1 was used. The internal consistency for the total scale was excellent (Cronbach’s α = 0.91).

Trait Anger Scale (TAS). Trait anger was assessed with the Korean adaptation (Chon et al., 1998) of the 15-item Spielberger et al. (1983) scale. To harmonize the measures, it was administered on a 5-point scale (α = 0.87).

Rosenberg Self-Esteem Scale (RSES). Global self-esteem was measured with the 10-item Korean version (Bae et al., 2014) on a 5-point scale (e.g., “On the whole, I am satisfied with myself”) (α = 0.89).

General Self-Efficacy Scale. Self-efficacy was assessed with the Korean version (Hong, 1995) of the Sherer et al. (1982) scale (e.g., “When I make plans, I am certain I can make them work”) (α = 0.86).

Life Orientation Test–Revised (LOT-R). Dispositional optimism/pessimism was measured with LOT-R (Scheier et al., 1994) (e.g., “In uncertain times, I usually expect the best”). Internal consistency was α = 0.78.

Beck Depression Inventory (BDI). Depressive symptoms were assessed using the 21-item Korean BDI (Lim et al., 2011), which showed α = 0.98 in this sample.

Trait Meta-Mood Scale (TMMS). To assess individual differences in attending to, clarifying, and repairing moods, we used a modified Korean translation (Lee & Lee, 1997) of the original TMMS (Salovey et al., 1995). The scale includes three subscales: Attention (e.g., “I pay a lot of attention to how I feel”), Clarity (e.g., “I am usually clear about my feelings”), and Repair (e.g., “I believe I can snap out of a bad mood”). All items were rated on a 5-point scale.

Emotion Regulation Checklist. Emotion regulation tendencies were assessed with the 12-item measure by Min et al. (2000), which yields Active (e.g., “I try to think of a way to solve the problem”), Avoidant (e.g., “I just assume things will somehow get better”), and Repressive subscales (e.g., “I try to completely block the problem out of my mind”).

### 3.2. Results

#### 3.2.1. Confirmatory Factor Analysis of the TSS

To confirm the three-factor structure of the Trait Sadness Scale (TSS) identified in Study 1, we conducted a confirmatory factor analysis (CFA) using the independent general population sample (*N* = 624). The analysis was performed using AMOS 26 with the maximum likelihood estimation method.

The hypothesized model specified three correlated latent factors—Affective Frequency, Cognitive Response, and Somatic/Behavioral Expression—with the 15 items loading onto their respective factors as determined by the EFA in Study 1. Model fit was evaluated using the chi-square statistic (*χ*^2^), the comparative fit index (CFI), the Tucker–Lewis index (TLI), the root mean square error of approximation (RMSEA), and the standardized root mean square residual (SRMR).

The three-factor model demonstrated a good fit to the data: *χ*^2^ (87) = 268.33, *p* < 0.001; *CFI* = 0.96; *TLI* = 0.95; *RMSEA* = 0.058 (90% CI = [0.050, 0.066]); and *SRMR* = 0.045. All standardized factor loadings were significant and substantial, ranging from 0.55 to 0.89. These results provide strong, independent support for the proposed three-factor structure of the TSS.

#### 3.2.2. Relationships with Personality and Emotion-Regulation Variables

To establish the TSS’s construct validity, its correlational profile with key personality and emotion regulation variables was examined, contrasting it with that of the TAS. Prior to analysis, we confirmed that the distributions of scale scores did not significantly deviate from normality, justifying the use of Pearson’s correlation coefficients.

Bivariate Correlations. To examine the personality profiles of the two emotional dispositions, their correlations with key personality and emotion regulation variables were calculated. A Bonferroni correction was applied to control for multiple comparisons across this family of 10 tests, setting the significance threshold at *p* < 0.005.

As hypothesized, the TSS was significantly and negatively associated with self-esteem (*r* = −0.48, *p* < 0.001), optimism (*r* = −0.40, *p* < 0.001), emotional clarity (*r* = −0.32, *p* < 0.001), and mood repair (*r* = −0.55, *p* < 0.001). It was also strongly and positively correlated with depression (*r* = 0.59, *p* < 0.001), avoidant coping (*r* = 0.12, *p* = 0.003), and repressive coping (*r* = 0.14, *p* = 0.001).

In contrast, the pattern for the TAS was sparser. Trait anger remained significantly correlated with optimism (*r* = −0.14, *p* < 0.001), depression (*r* = 0.24, *p* < 0.001), self-efficacy (*r* = −0.12, *p* = 0.002), mood repair (*r* = −0.17, *p* < 0.001), and avoidant coping (*r* = 0.12, *p* = 0.002). This initial pattern suggests that while both traits share some overlap in the domain of negative affectivity, the broad profile of diminished self-appraisals and specific emotion regulation deficits is more characteristic of a sad disposition.

Partial Correlation Analysis. To statistically disentangle the unique correlates of trait sadness and trait anger, partial correlations were computed, again applying a Bonferroni-corrected threshold of *p* < 0.005. The results revealed a clear asymmetry.

When controlling for trait anger, the strong correlations between trait sadness and the personality variables remained virtually unchanged. TSS retained significant unique associations with self-esteem (*r* = −0.47, *p* < 0.001), optimism (*r* = −0.39, *p* < 0.001), depression (r = 0.55, *p* < 0.001), self-efficacy (*r* = −0.48, *p* < 0.001), emotional clarity (*r* = −0.34, *p* < 0.001), and mood repair (*r* = −0.38, *p* < 0.001).

Conversely, when controlling for trait sadness, all significant correlations between trait anger and the personality variables disappeared. None of the associations met the corrected significance threshold. This pattern provides powerful evidence that the observed personality profile—lower self-esteem, optimism, and mood repair, coupled with higher depression—is specific to trait sadness. The associations for trait anger appear to be largely an artifact of its shared variance with trait sadness, rather than a unique effect of trait anger itself.

## 4. Study 2: Emotional Disposition, Cognitive Appraisal, and Helplessness

The primary aim of Study 2 was to experimentally investigate how emotional dispositions—specifically sadness-proneness versus anger-proneness—shape appraisals of ambiguous social situations. By presenting participants with scenarios where the cause of a negative outcome was unclear, this study sought to test whether these two groups would exhibit distinct attributional patterns (judgments of cause) and differ in their helplessness appraisals.

Cognitive appraisal theories and the Appraisal Tendency Framework (ATF) posit that transient state emotions bias causal attribution; sadness typically promotes appraisals of situational factors, whereas anger promotes appraisals of other-person factors (i.e., blame). This study tests whether this well-established link extends from momentary states to stable trait dispositions. We hypothesized that a history of frequent sad or angry experiences would make the corresponding attributional styles chronically accessible.

A second key dimension distinguishing these emotions is the appraisal of personal control and the potential for goal restoration. Research demonstrates that anger is typically experienced when a blocked goal is seen as recoverable, whereas sadness follows when the goal is appraised as irrevocably lost (Levine, 1995). Extrapolating from this, it was hypothesized that sadness-prone individuals would report greater helplessness than their anger-prone counterparts, particularly in response to negative outcomes.

Taken together, Study 2 was designed to experimentally test two central hypotheses: that the distinct cognitive signatures of state sadness and anger—namely, situational attribution and appraisals of helplessness—are also stable features of individuals with a dispositional tendency toward these respective emotions.

### 4.1. Method

#### 4.1.1. Participants

The final experimental sample consisted of 244 undergraduate students (136 men and 108 women) recruited from three universities in South Korea. Participants provided written informed consent before the initial screening. The mean age of participants was 21 years (*SD* = 2.4).

#### 4.1.2. Procedure

The study was conducted in two phases. First, an initial screening questionnaire containing the Trait Sadness Scale (TSS), Trait Anger Scale (TAS), and Beck Depression Inventory (BDI) was administered to 630 respondents.

From this pool, two distinct emotional disposition groups were created using a median-split procedure, a validated method for maximizing statistical power when comparing distinct personality profiles (e.g., Deffenbacher et al., 1988). Participants scoring above the median on the TSS and below the median on the TAS were categorized as the Sadness-Prone Group. Conversely, those scoring above the median on the TAS and below the median on the TSS were categorized as the Anger-Prone Group. While this approach reduces the full range of trait scores to a categorical variable, it is a powerful method for isolating and contrasting the distinct cognitive signatures of individuals who predominantly experience one negative emotion over the other.

This procedure identified 121 sadness-prone and 123 anger-prone individuals who were invited to participate in the main scenario-based experiment. Participants were instructed to read two vignettes and completed a cognitive judgment questionnaire after each.

#### 4.1.3. Measures

The measurement strategy for Study 2 was twofold. For the immediate post-scenario judgments, we intentionally used a simple, forced-choice format to capture participants’ most accessible, primary “gut” reaction, an approach consistent with the predictions of the Appraisal Tendency Framework. To provide a more nuanced, supplementary assessment of attributional style, we also included the open-ended Life Events Attribution Questionnaire (LEAQ) during the initial screening.

Ambiguous Scenarios. Two scenarios depicting negative outcomes with ambiguous causes were used: a failed job interview (achievement context) and a romantic breakup (relationship context). These vignettes were designed to be realistic and relatable for undergraduate students. These were selected from a larger pool after pilot testing confirmed they elicited a balanced range of both sadness and anger responses. The full text for each scenario is provided in Appendix A.

Cognitive Judgment Questionnaire. Following each scenario, participants completed a questionnaire assessing three components: (1) Emotional response: Participants rated how they would feel on a 7-point scale using six emotion words: angry, discouraged, annoyed, depressed, irritated, and sad. They then selected the single word that best described their feeling. This primary selection was coded as Anger (angry, annoyed, and irritated) or Sadness (discouraged, depressed, and sad). (2) Causal attribution: Participants answered “What do you think was the most important cause of this event?” by choosing one of four options, which were classified as Self (e.g., “It was something I did or didn’t do”), Other (e.g., “It was the interviewer’s fault”), or Situation (e.g., “It was just bad luck” or “Nobody was really at fault”). (3) Helplessness: Participants rated their agreement with the statement “There is nothing more I can do in this situation,” on a 9-point scale from 1 (not at all) to 9 (absolutely).

Life Events Attribution Questionnaire (LEAQ). Participants also completed the LEAQ. For several hypothetical negative events, they were asked to generate the primary cause for each event and then rate it along three dimensions of attributional style: Locus (internal vs. external), Stability (a stable, recurring cause vs. an unstable, one-time cause), and Globality (a global cause affecting many areas of life vs. a specific cause). For example, one item stated “I had a quarrel with family members.” Attribution scores for each dimension were averaged across the four cue items.

### 4.2. Results

#### 4.2.1. Preliminary Analyses

Before testing the main hypotheses, preliminary analyses were conducted. The final sample of 244 participants was evenly split between the Sadness-Prone Group (*n* = 121) and the Anger-Prone Group (*n* = 123). A chi-square analysis revealed a significant association between emotional disposition and gender; the anger-prone group included more men, while the sadness-prone group included more women, *χ*^2^(1, *N* = 244) = 12.01, *p* < 0.001, Cramér’s *V* = 0.22.

A manipulation check confirmed that the groups responded to the scenarios as expected. Across both scenarios, sadness-prone individuals reported significantly more sadness than anger, while anger-prone individuals reported significantly more anger than sadness (Achievement: *χ*^2^(1, *N* = 244) = 4.817, *p* < 0.001; Relationship: *χ*^2^(1, *N* = 244) = 12.967, *p* < 0.001). This confirms that their emotional dispositions biased their in-the-moment reactions in the predicted direction.

In a supplementary analysis to establish discriminant validity from depression, it was found that a participant’s depression level (high vs. low on the BDI) did not predict their emotional response to the scenarios. Unlike the emotional disposition groups, the high-depression group did not report significantly more sadness than the low-depression group in the achievement scenario *χ*^2^(1, *N* = 244) = 0.962, *p* = 0.368 as well as in the relationship scenario *χ^2^*(1, *N* = 244) = 0.017, *p* = 0.935. This supports the conclusion that trait sadness is a construct distinct from depression.

#### 4.2.2. Replication of State-Level Emotion–Attribution Links

**Figure 1:**
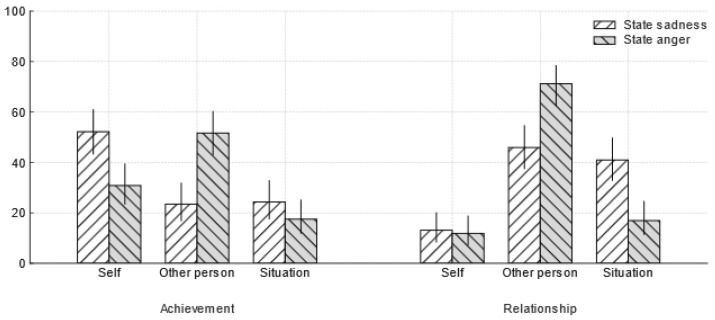
Attribution patterns by scenario as a function of state emotion. Bars show the percentage of attributions within each emotion group (state sadness vs. state anger) to Self, Other person, or Situation in the Achievement and Relationship scenarios. Error bars are 95% Wilson score confidence intervals for the within-group proportions.

To validate our experimental paradigm, the link between state emotional responses and causal attributions was first tested. Consistent with classic cognitive appraisal theory, there was a significant association between state emotion and causal attributions in both scenarios. In the achievement scenario, participants reporting state sadness made more self-attributions, whereas those reporting state anger made significantly more other-person attributions, *χ*^2^(2, *N* = 244) = 22.45, *p* < 0.001, Cramér’s *V* = 0.30. In the relationship scenario, participants reporting state sadness tended to make other-person or situational attributions, whereas those reporting state anger made predominantly other-person attributions, *χ*^2^(2, *N* = 244) = 18.53, *p* < 0.001, Cramér’s *V* = 0.28 (See Figure 1). This successfully replicates previous findings and confirms the paradigm’s sensitivity.

#### 4.2.3. Emotional Disposition and Locus of Attribution

**Figure 2:**
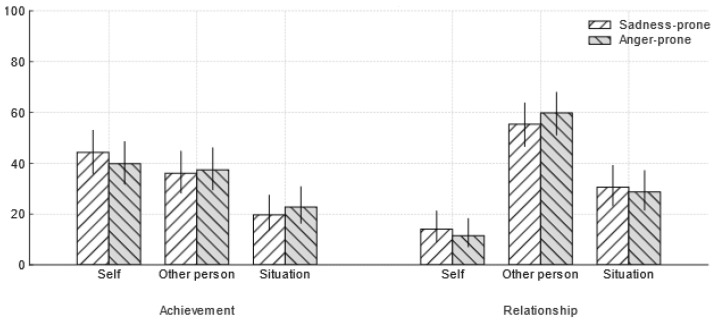
Attribution patterns by scenario as a function of emotional disposition. Bars show the percentage of attributions within each disposition group (sadness-prone vs. anger-prone) to Self, Other person, or Situation in the Achievement and Relationship scenarios. Error bars are 95% Wilson score confidence intervals for the within-group proportions.

The central hypothesis predicted that sadness-prone and anger-prone groups would differ in their primary causal attributions. This hypothesis was not supported. A chi-square analysis revealed no significant association between emotional disposition toward sadness versus anger and locus of attribution (Self vs. Other vs. Situation) in either scenario. In the achievement scenario, sadness-prone participants most often made self-attribution, followed by other and situation attribution; anger-prone participants showed the same pattern, *χ*^2^(2, *N* = 244) = 0.59, *p* = 0.74, Cramér’s *V* = 0.049. In the relationship scenario, sadness-prone participants most often made other-attribution followed by situation and self-attribution; similarly anger-prone participants most often made other-attribution followed by situation and self-attribution, *χ*^2^(2, *N* = 244) = 0.60, *p* = 0.74, Cramér’s *V* = 0.050. As shown in Figure 2, the distributions across disposition groups were broadly similar, and the observed differences were not statistically reliable.

#### 4.2.4. Emotional Disposition and Helplessness

**Figure 3:**
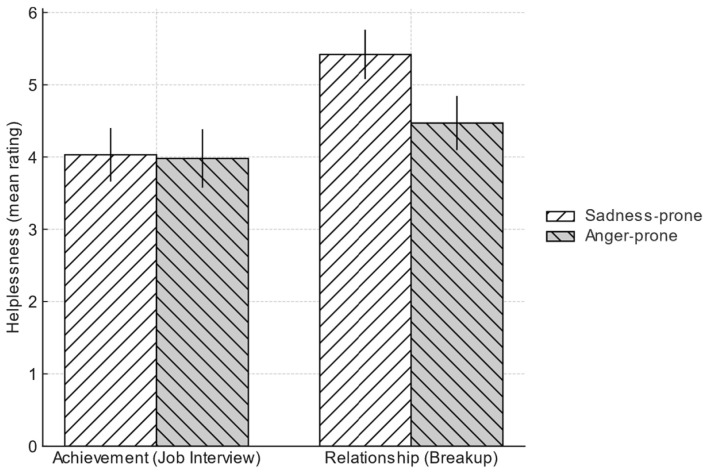
Helplessness by scenario for sadness-prone and anger-prone groups; error bars are 95% t-based confidence intervals.

The second hypothesis predicted that the sadness-prone group would experience greater helplessness. The results supported this prediction but revealed a critical context-dependency. In the achievement-oriented scenario, there was no significant difference in helplessness ratings of sadness-prone group (*M* = 4.03, *SD* = 2.06) and anger-prone group (*M* = 3.98, *SD* = 2.27), *t*(242) = 0.18, *p* = 0.861, Cohen’s *d* = 0.02. In the relationship-oriented scenario, the hypothesis was strongly supported. The sadness-prone group reported significantly greater feelings of helplessness (*M* = 5.42, *SD* = 1.9) than the anger-prone group (*M* = 4.47, *SD* = 2.1), *t*(242) = 2.97, *p* = 0.003, Cohen’s *d* = 0.38 (See Figure 3). This effect remained significant when controlling for participants’ gender and BDI scores in a follow-up ANCOVA, indicating the finding is not an artifact of these variables. This suggests that the heightened sense of helplessness core to a sad disposition is not uniform but is particularly pronounced in the context of interpersonal loss and rejection.

#### 4.2.5. Follow-Up Analysis of Attributional Style (LEAQ)

**Figure 4:**
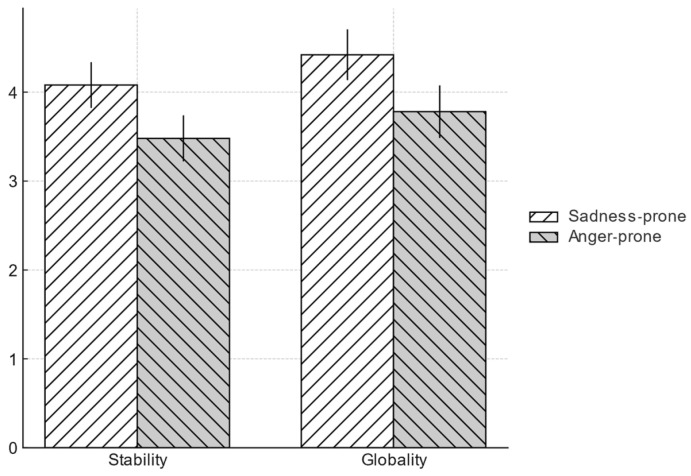
Stability and globality by emotional disposition. Bars show group means for sadness-prone and anger-prone; error bars are 95% t-based CIs around each mean.

To further explore attributional differences using a method distinct from the scenario results, data from the Life Events Attribution Questionnaire were analyzed. Consistent with the scenario study, no significant differences between sadness-prone and anger-prone individuals were observed for the locus of attribution in either achievement or relationship contexts. However, clear group differences emerged for stability and globality. For stability, sadness-prone participants (*M* = 4.08, *SD* = 1.43) rated the causes of negative events as more stable than did anger-prone participants (*M* = 3.48, *SD* = 1.45), *t*(242) = 3.25, *p* = 0.001, *d* = 0.42, 95% CI [0.24, 0.96]. For globality, sadness-prone participants (*M* = 4.42, *SD* = 1.59) also rated causes as more global than did anger-prone participants (*M* = 3.78, *SD* = 1.66), *t*(242) = 3.08, *p* = 0.002, *d* = 0.39, 95% CI [0.23, 1.05] (see Figure 4). These effects for both stability and globality remained significant even after controlling for participants’ gender and BDI scores in follow-up ANCOVAs.

Taken together, these findings suggest that the key cognitive difference between the two dispositions may not be who or what they blame, but rather their perception of the cause’s pervasiveness and chronicity.

### 4.3. Discussion

This study yielded a nuanced set of results. As expected, participants’ emotional dispositions predicted their state reactions to ambiguous events, and these state reactions were linked to attributions in a manner consistent with classic appraisal theory. However, the study’s central hypothesis was not supported: the link between emotion and locus of attribution did not extend to the trait level. Despite showing distinct emotional reactions, sadness-prone and anger-prone individuals did not differ in who or what they blamed for negative outcomes. In contrast, the hypothesis regarding helplessness was partially supported, with sadness-prone individuals reporting greater helplessness specifically within an interpersonal context.

The observation of a strong link between state emotion and attribution alongside a null link at the trait level is the study’s most critical finding. It challenges a direct extension of state-level appraisal models to emotional dispositions and suggests that locus of attribution may not be the primary mechanism differentiating these temperaments. The data imply that sadness-prone individuals default to sadness and anger-prone individuals default to anger, even when their initial causal interpretations of an event are nearly identical.

If not locus of attribution, what drives these temperamental differences? Our follow-up analyses provide a clue. The finding that sadness-prone individuals appraise causes as more stable and global suggests the key cognitive difference lies in the perceived pervasiveness and chronicity of negative events. This pessimistic explanatory style, rather than simple blame, may be the cognitive signature of trait sadness.

Furthermore, the context-dependent nature of the helplessness finding is highly informative. The fact that differences only emerged in the relationship scenario suggests that feelings of powerlessness tied to trait sadness are most salient in the interpersonal domain, a finding that aligns with the established role of sadness in signaling the loss of social bonds.

A limitation of this study is its use of single scenarios for each context. Future research should use a wider array of scenarios to confirm the robustness of the context-dependent helplessness effect. Additionally, while our data point toward alternative cognitive mechanisms, other factors, such as biological predispositions (e.g., baseline physiological arousal), warrant investigation. Integrating biological measures with the cognitive approach used here represents a promising avenue for future research.

In conclusion, these findings suggest that dispositions like sadness- and anger-proneness are not simply chronic versions of their state-level counterparts. They are differentiated less by who is blamed and more by context-dependent feelings of control and a pervasive, pessimistic view of negative life events.

## 5. General Discussion

This research began with the goal of delineating the cognitive profiles of trait sadness and trait anger. Its central aim was to test the widespread assumption that the cognitive appraisals differentiating emotional states (e.g., causal attribution) would also differentiate their corresponding emotional traits. The findings, however, challenge this assumption, revealing a dissociation between state- and trait-level cognition and pointing toward alternative mechanisms that define these enduring emotional styles.

Across two studies, the Trait Sadness Scale (TSS)—a three-factor measure assessing affective frequency, cognitive responses, and somatic/behavioral expressions of sadness —was first developed and validated (Study 1). This new instrument enabled a direct comparison of sadness-prone and anger-prone individuals. Contrary to our primary hypothesis, the two groups did not differ in their causal attributions for negative events—a null finding replicated across both scenario-based and questionnaire methods. Instead, key differences emerged elsewhere: sadness-prone individuals reported greater helplessness in interpersonal contexts and appraised negative events as more stable and global in nature.

### 5.1. The Central Finding: A State–Trait Dissociation

The core finding of this research is the divergence between emotion–cognition links at the state versus the trait level. While the paradigm used in this study successfully replicated the classic finding that state sadness and anger are tied to distinct attributional patterns, this relationship vanished when examining dispositions. This suggests that a person’s habitual attributional style for who to blame may not be the foundational mechanism that gives rise to a sad or angry temperament.

This finding adds critical nuance to the literature. For instance, Lerner and Keltner (2001) found that the link between fear/anger and risk perception did extend from the state to the trait level. The present results, by failing to find a similar extension for sadness/anger and causal attribution, suggest that the correspondence between state- and trait-level cognition is not a universal rule but is likely specific to certain emotion-cognition pairings.

### 5.2. Implications and Alternative Mechanisms

If locus of attribution is not the primary driver, what differentiates these emotional dispositions? The present data point to two promising avenues. The first is the appraisal of personal control in specific contexts. The finding that sadness-prone individuals felt more helpless, but only in an interpersonal scenario, suggests that their disposition may be characterized by a particular vulnerability to social relational powerlessness. The second is attributional style, but in a manner different from what was initially hypothesized. The key distinction was not in locus of causality (self vs. other) but in the perceived chronicity and pervasiveness of negative events—that is, in stable and global attributions. This pessimistic explanatory style, rather than blame, may be the true cognitive signature of trait sadness.

Beyond these cognitive factors, another plausible explanation lies in biological predispositions. Individuals with lower baseline physiological arousal may be more inclined toward low-arousal sadness, while those with higher arousal may default to high-arousal anger. Future research employing multi-method approaches that combine cognitive measures with physiological assessments (e.g., Gabbay, 1992) is essential to test these competing mechanisms.

### 5.3. Distinguishing Trait Sadness from Depression

A unique contribution of this work is its empirical differentiation of trait sadness from depression. A propensity for sadness is often treated as equivalent to subclinical depression (Bondolfi et al., 2015), yet this is rarely verified empirically. Our finding that trait sadness, but not BDI scores, predicted a specific bias toward sad feelings in ambiguous scenarios provides strong evidence that they are separate constructs. This suggests that subclinical depression encompasses more complex factors than a mere vulnerability to sadness. Future research should continue to delineate the emotional characteristics of subclinical depression to clarify the similarities and differences between these two important dispositions.

### 5.4. Practical Implications

Beyond its theoretical contributions, this research holds significant practical implications, primarily through the development and validation of the Trait Sadness Scale (TSS). In clinical and counseling settings, the TSS can serve as a valuable tool to help clients identify and understand their core emotional patterns. As demonstrated in this research, the ability to empirically distinguish a disposition for sadness from clinical depression is crucial; it allows clinicians to tailor interventions more precisely to an individual’s specific affective profile, rather than applying a one-size-fits-all approach.

As a research instrument, the TSS opens new avenues for exploring the downstream consequences of trait sadness. Its application could extend into health psychology to investigate links between this disposition and physical well-being, into developmental psychology to track how emotional traits evolve over the lifespan, and into organizational psychology to better understand its impact on employee resilience and workplace dynamics. By providing a reliable measure for a fundamental emotional trait, the TSS equips researchers to more accurately map the role of sadness in shaping human experience.

### 5.5. Limitations and Future Directions

While the present research provides a strong foundation, several limitations should be noted. First, Studies 1-1 and 2 relied on undergraduate student samples. While appropriate for initial scale development and testing theoretical links, their demographic homogeneity may limit the generalizability of some findings. Similarly, our general population sample in Study 1-2 was skewed toward younger adults. Future research should therefore aim to validate the TSS in older and more occupationally diverse community samples.

Second, although our follow-up measurement invariance analysis confirmed that the TSS’s three-factor structure holds across genders, the initial EFA sample had an unequal gender distribution. The question of whether mean levels of trait sadness differ between genders, and how social norms shape its expression, remains a fruitful avenue for future investigation.

Third, the experimental design in Study 2 relied on a single vignette for each of the achievement and relationship contexts. While these scenarios were carefully pilot-tested, future research should employ a wider array of situations to confirm the robustness of the context-dependent helplessness effect and to ensure the findings are not an artifact of the specific narratives used.

## 6. Conclusions

This research developed and validated a new tool for measuring trait sadness and used it to challenge an assumption about the nature of emotional dispositions. It demonstrates that the cognitive architecture of emotional states does not simply scale up to define emotional traits. Instead, it points toward a more complex picture where emotional dispositions are defined by a nuanced interplay of context-dependent appraisals of control, a pessimistic explanatory style, and potentially underlying biological factors.

## Figures and Tables

**Table 1:** Pattern Matrix from the Exploratory Factor Analysis for the Final 15-Item TSS (*N* = 161).

Item	Factor 1 (Affective)	Factor 2 (Cognitive)	Factor 3 (Somatic/Behav.)
I get depressed easily.	**0.85**	0.01	0.04
I often feel sad in my daily life.	**0.71**	0.08	0.12
I often feel helplessness.	**0.58**	0.26	−0.03
I often get dejected.	**0.51**	0.28	0.04
I feel unfortunate.	**0.49**	0.29	−0.02
When things go off course, I feel it’s hard to set them right again on my own.	−0.01	**0.78**	0.06
After a setback, I feel like I can’t cope.	0.02	**0.71**	0.06
I find it hard to snap out of it when I’m feeling sad.	0.23	**0.64**	0.17
I am saddened by the gap between how things are and how I want them to be.	0.24	**0.55**	0.23
Frustration makes me feel drained and want to quit.	0.08	**0.48**	0.25
When someone rejects me, I feel like crying.	−0.06	0.11	**0.71**
I get a lump in my throat when I’m ignored.	0.01	0.01	**0.71**
Sadness feels like a wrenching pain in my chest.	0.03	0.13	**0.69**
There are times when I get a sudden urge to cry.	0.22	−0.17	**0.63**
When I’m sad, it feels hard to even move a finger.	0.08	0.12	**0.52**

Note. Primary factor loadings are in bold. Item wording has been abbreviated for clarity.

**Table 2:** Zero-Order Correlations Among TSS and DES-IV Subscales.

DES Subscale	Joy	Interest	Sadness	Contempt	Surprise	Fear	Anger
*Pearson r*	−0.43	−0.31	0.74	0.17	0.27	0.46	0.55
*Sig.*	0.000	0.000	0.000	0.035	0.001	0.000	0.000

Note. A Bonferroni-corrected significance threshold of *p* < 0.007 was applied to the DES subscale correlations. The *p*-value for Contempt (*p* = 0.035) exceeded this threshold.

## Data Availability

Data can be obtained upon request from the corresponding author.

## Appendix A. Full Text of Scenarios Used in Study 2

**Achievement-Oriented Scenario**

As a fourth-year student on the verge of graduation, you’ve seen many upperclassmen struggle to find jobs, so you started preparing early. You’ve achieved a high level of proficiency in both English and Japanese, recently earned a professional certification, and maintained a strong GPA.

In your final year, you identified a company that seemed like a perfect fit. The more you learned, the more certain you were that you wanted to work there. An alumnus with connections to the HR team gave you some inside information: the company values objective qualifications (like language proficiency and grades) and prefers candidates who are cooperative team players over those who try to stand out.

Confident that you were an excellent match, you passed the initial screening rounds and advanced to the final interview. On the day of the interview, you prepared meticulously, wearing a formal suit and rehearsing your answers. In the interview room, you were joined by just one other candidate, who had a noticeably free-spirited demeanor.

The interviewers asked you both to discuss a familiar topic. You presented your views clearly and logically, just as you had prepared. The other candidate’s responses, however, were highly original—unconventional, yet you had to admit, persuasive in their own way. While most of the panel seemed to favor your approach, one influential interviewer was a clear exception. This person showed strong interest in the other candidate’s remarks and criticized your answers as being “too formulaic”.

A few days later, you receive the results: the other candidate was selected. As the disappointing news sinks in, your mind keeps replaying that one interviewer’s sharp criticism, leaving you with a mix of confusing feelings, unsure of what went wrong.

**Relationship-Oriented Scenario**

You and your close friends are hosting an end-of-semester, student-run pop-up pub near campus and have invited people to drop by. Among those you invited is someone you have been interested in throughout the term. They have seemed interested in you as well and readily accepted your invitation. Because you find this person very attractive, you feel excited by their positive response.

As the day approaches, you worry they might feel awkward at the event, so you tell your friends about them and ask your friends to be especially welcoming. On the day of the event, you spend time deciding what to wear and how to do your hair, feeling hopeful.

Most friends arrive on time and the atmosphere warms up, but you keep glancing toward the entrance, wondering when this person will appear. When the door chime rings, you hurry to the counter. You see the person arrive with their partner and smiling in your direction. Their partner is well known at your school and widely considered very attractive.

A brief silence falls among your friends who had been watching. Then you hear whispering: “Of all people, you ended up liking someone who already has a partner… Just because someone is taken doesn’t mean nothing can happen, but still—that’s unfortunate.” Those words linger, and you start to feel as if everyone can tell how you feel about this person. After a moment, you look around and notice the person laughing affectionately with their partner, seemingly unaware of your presence.
